# Self-Calibration and Performance Control of MEMS with Applications for IoT

**DOI:** 10.3390/s18124411

**Published:** 2018-12-13

**Authors:** Jason Clark

**Affiliations:** Electrical and Computer Engineering, Auburn University, Auburn, AL 36830, USA; jvclark@auburn.edu

**Keywords:** metrology, feedback, self-calibration, performance control

## Abstract

A systemic problem for microelectromechanical systems (MEMS) has been the large gap between their predicted and actual performances. Due to process variations, no two MEMS have been able to perform identically. In-factory calibration is often required, which can represent as much as three-fourths of the manufacturing costs. Such issues are challenges for microsensors that require higher accuracy and lower cost. Towards addressing these issues, this paper describes how microscale attributes may be used to enable MEMS to accurately calibrate themselves without external references, or enable actual devices to match their predicted performances. Previously, we validated how MEMS with comb drives can be used to autonomously self-measure their change in geometry in going from layout to manufactured, and we verified how MEMS can be made to increase or decrease their effective mass, damping, and or stiffness in real-time to match desired specifications. Here, we present how self-calibration and performance control may be used to accurately sense and extend the capabilities of a variety of sensing applications for the Internet of things (IoT). Discussions of IoT applications include: (1) measuring absolute temperature due to thermally-induced vibrations; (2) measuring the stiffness of atomic force microscope or biosensor cantilevers; (3) MEMS weighing scales; (4) MEMS gravimeters and altimeters; (5) inertial measurement units that can measure all four non-inertial forces; (6) self-calibrating implantable pressure sensors; (7) diagnostic chips for quality control; (8) closing the gap from experiment to simulation; (9) control of the value of resonance frequency to counter drift or to match modes; (10) control of the value of the quality factor; and (11) low-amplitude Duffing nonlinearity for wideband high-Q resonance.

## 1. Introduction

Attributes of microelectromechanical systems (MEMS) include a wide variety of transduction capabilities in packages that are small in size, weight, power, and cost. Such attributes facilitate the Internet of Things (IoT), where physically-sensed information about the environment is passed between objects (things) and the Internet. In addition to monitoring the performance of critical processes, MEMS that are widely and ubiquitously distributed will enable the Internet to sense the physical world. Examples of IoT application areas include: (1) society—health care, media and entertainment, smart environments, intelligent transportation, smart retail, security and surveillance; (2) environment—smart agriculture, disaster management, pollution control, and smart power plants; and (3) industry—supply chain management, aerospace and aviation, transportation and logistics, smart metering, warehouse and storage [1,2,3,4]. IoT will generate an enormous amount of data. The quality of sensed data will be based on the performance of MEMS. Although MEMS are very precise, they have accuracy issues.

There are many IoT applications that do to not require MEMS sensors with high accuracy, precision, or performance control, but for present or future applications that cannot afford wide margins of uncertainty, then highly accurate, precise, and extended behavioral control may make such applications possible. For instance, the inability of today’s MEMS to self-calibrate means that a distribution of MEMS will have a large uncertainty and unknown accuracy. Inaccurate data fed into an accurate model will yield inaccurate results. That is, while a 10% uncertainty can be sufficient for some applications, it may not be sufficient for others, and for applications that require a distribution of MEMS to behave identically, or to match prediction, or to change behavior altogether, such systems may greatly benefit from performance control technology.

For envisioned IoT applications that require accurate sensing or well-specified performance, advances in calibration or control for MEMS during deployment is needed. Due to systemic process variations, increasing design complexities, and increasing integration, the International Technology Roadmap for Semiconductors (ITRS) identifies packaging and testing as the greatest challenges for microelectromechanical systems (MEMS) technologies [5,6,7]. An inertial measurement unit (IMU) is one example of an integrated system, consisting of a tri-axis accelerometer (translation), tri-axis gyroscope (rotation), tri-axis magnetometer (compass), and pressure sensor (altimeter), where the greatest challenge is improving resolution, bias, and drift while reducing costs.

There has not been a quick and inexpensive method to test such 10 degree of freedom (DOF) multimode sensors at the wafer- or chip-level, which bottlenecks throughput, increases cost, and impedes design improvements. Such difficulties require designers to consider the back-end of manufacturing issues, such as packaging and testing, at the beginning of the design process. In-factory testing of devices amounts to 25% to 75% of manufacturing costs [5].

The MEMS community is also lacking a comprehensive set of measurement standards. Although there are plenty of testing methods, there are too few testing standards. Standards are needed because different testing methods yield different measurement results, which discourages a consensus on preferred methods. The lack of standards creates problems at hand-off points between processing stages, between manufacturers, and it negatively affects international commerce. Currently, there are standards for measuring Young’s modulus, cantilever length, layer thickness, and residual stress/strain [8]. However, the standards themselves can have issues. For instance, the Young’s modulus standard requires the measurement of density (which has no microscale standard), thickness (where profilometry is affected by surface roughness and underlying gap, and ellipsometry is affected by material transparency and spot size which is typically much larger than flexure width), undamped out-of-plane resonance (many MEMS deflect in-plane, and many materials are anisotropic), anchor type (boundary compliance and fillets measurably affect performance), and actuation method (affects mode shape). Most measurement methods are functions of one or more quantities that do not have a standard, are not well-measured, or are found in a look-up table. Such issues yield large uncertainties (>10%) and unknown accuracy.

A 10% error results in a measurement value having just one significant digit. Inaccurate data can result in misinformed decisions. Although some IoT applications will not require measurements beyond one significant digit, there are many IoT applications envisioned that will need to correctly measure small subtleties, be able to recalibrate after long-term dormancy or after harsh environmental changes, and be able to bridge the large gap between measurement and corresponding predictive models.

Regarding process variations, geometric and material properties measurably vary between facilities, between successive runs at the same facility, and between chips on the same wafer. Device packaging, which can also affect performance, often needs to be considered throughout all stages of sensor design. Small variations can significantly affect performance. For instance, a quarter-micron overcut (near the diffraction limit of visible light) on a flexure that is 2 µm in width will result in planar stiffness that is nearly twice as large as was predicted by its layout geometry.

Due to the numerous design parameters and the issues with measuring them, a “black box” method is often employed in the factory to calibrate MEMS, where output signals are correlated with input disturbances. In addition to the abovementioned problems of testing multi-DOF systems, since the equation of motion of the system remains unknown, it is possible for output signals due to untested combinations of disturbances to incorrectly identify causes. Unlike the electrical domain, where inductance, resistance, capacitance, voltage, and current are readily measured and traceable to international measurement standards, there have not been accurate and traceable ways to measure microscale mass, damping, stiffness, displacement, and force for MEMS.

In addition to efforts for measuring manufactured geometric and material properties, there have been efforts to compensate for such process variations such as post-fabrication mechanical or electrical tuning.

Examples of mechanical tuning include methods that remove material to adjust mass or stiffness such as laser trimming [9] or reactive ion etching or ion milling [10], and methods that add material such as polysilicon deposition [11] or silver electro-deposition [12]. Such methods were shown to help compensate for process variation by adjusting resonant frequency by 10%.

Prior efforts in electrical tuning include methods to improve quality factor, adjust stiffness, and modify effective damping. For instance, in Reference [13], quality factor was increased three orders by increasing the effective stiffness of a cantilever through position-controlled feedback. In Reference [14], position-controlled force-feedback used to improve bandwidth and linearity. In Reference [15], tapered comb fingers under a DC bias reduce resonant frequency. In Reference [16], position-controlled digital force-feedback was used to modify effective stiffness to reduce resonance frequency by 93%. In References [17,18,19], effective damping was reduced through velocity-controlled electrical feedback. And in Reference [20], tunable bifurcation was demonstrated in a linear system using analog electronic feedback spring softening and hardening.

Towards the self-calibration and performance control of MEMS for IoT applications, this paper describes: (1) how mass, damping, stiffness, and force of a MEMS device can be accurately measured with quantifiable uncertainty in Section 2; (2) how such quantities can be changed on demand in Section 3; and (3) how such capabilities may benefit IoT in Section 4. We summarize in Section 5.

## 2. Self-Calibration

This section describes how packaged and deployed MEMS devices with comb drives can be made to accurately measure their own mass, damping, stiffness, displacement, and force without the need for in-factory calibration or external references. The extension of this metrology to other types of MEMS sensors is discussed below. The method is accurate, repeatable, and reliable. Here, accuracy is a measure of the difference between the average value and the true value; uncertainty is the order of the most significant uncertain digit due to the totality of measurement noise; repeatability is the ability of a measurement method to obtain the same value after reassembling the experiment; and reliability is the ability of the same measurement method to achieve the same results by using different testing equipment (i.e., test equipment by a different manufacturer). We define verification as a test of how well different methods of analyses agree with each other (such as analytical theory versus simulation); and we define validation as a test of how well a measurement method agrees with the true value.

*Validation*. The lack of accurate and traceable measurement standards for MEMS makes direct validation difficult because the true value is unknown. However, a condition that must hold true in the validation of any metrological method is that two or more sensors that use that method must agree on their measured values whenever identical disturbances are applied. For instance, although the amount of an applied force may be unknown, two different sensors that use the same metrological method to measure that force should agree on their measurements of that force.

*Assumptions*. We assume that: (1) Geometric features that are within close-proximity to each other undergo identical process variations. This assumption is, of course, measurable through validation tests. (2) The following analysis requires that comb drives operate within their linear range of motion.

*Sequence of measurements*. We previously presented the electrically-probed measurement of the gap between structures and flexure width. The measurements were validated against scanning electron microscopy [21,22]. In either case, a relationship was necessarily identified that expressed the mechanical quantity as a function of electrical measurands only. Doing so enables measurements to be performed completely within a packaged chip. Since most MEMS relations are coupled to each other by one or more other mechanical quantities, then a sequence of quantity extraction exists. That is, relationships between unknown mechanical quantities $m_{i}^{}$ (such as stiffness, width, mass, gap, damping, force, Young’s modulus, density, etc.) and measurable electrical quantities $e_{i}^{}$ (such as capacitance, voltage, frequency, etc.) can be expressed by the following electro-micro-metrology (EMM) functions $G_{i}^{}$:

(1) $$\begin{array}{l} m_{1}=G_{1}^{*}\left(e_{1}\right)\\ m_{2}=G_{2}\left(m_{1},e_{2}\right)=G_{1}\left(e_{1},e_{2}\right)\\ m_{3}=G_{3}\left(m_{2},e_{3}\right)=G_{2}\left(e_{1},e_{2},e_{3}\right)\\ \vdots \\ m_{N}=G_{N}\left(m_{N-1},e_{N}\right)=G_{N}\left(e_{1},\ldots ,e_{N}\right) \end{array}$$

where the first seed relation $G_{1}^{*}$ is special in that it is not a function of any mechanical quantity. All subsequent measurements of mechanical quantities $m_{i}^{},i>1$, depend on one or more prior measurements $m_{j}^{},j<i$. However, each $m_{i}^{}$ quantity is ultimately a function of electrical measurands only. In all, relations in Equation (1) form a ordered sequence of quantity extractions. Due to interdependency between mechanical quantities, a large number of mechanical quantities can be determined from a much smaller number of electrical measurands.

*Quantifiable uncertainty*. The uncertainty of an electrical measurand, $\delta e_{j}$, is easily determined by, for example, the order of the most significant uncertain digit on its readout meter. Subsequently, the uncertainty of the mechanical quantity, $\delta m_{i}$, of the MEMS device can be determined by a 1st-order multivariate Taylor expansion about $e_{j}$ as shown in Equation (2).

In Equation (2), the small-valued electrical uncertainties $\delta e_{j}$ are multiplied by the large-valued electromechanical sensitivities $\partial G_{i}/\delta e_{j}$. It is not uncommon to have uncertainties on the order of $O\left(10^{-18}\right)$ with corresponding sensitivities on the order of $O\left(10^{12}\right)$. As length scale decreases, so too does the sensitivity, which is a reason why this metrology method becomes tractable at the microscale and below:

(2) $$\begin{array}{l} \delta m_{1}=\frac{\partial G_{1}^{*}}{\partial e_{1}}\delta e_{1}\\ \delta m_{2}=\frac{\partial G_{2}}{\partial e_{1}}\delta e_{1}+\frac{\partial G_{2}}{\partial e_{2}}\delta e_{2}\\ \delta m_{3}=\frac{\partial G_{3}}{\partial e_{1}}\delta e_{1}+\frac{\partial G_{3}}{\partial e_{2}}\delta e_{2}+\frac{\partial G_{3}}{\partial e_{3}}\delta e_{3}\\ \vdots \\ \delta m_{N}=\frac{\partial G_{N}}{\partial e_{1}}\delta e_{1}+\ldots +\frac{\partial G_{N}}{\partial e_{N}}\delta e_{N}. \end{array}$$

**Figure 1:**
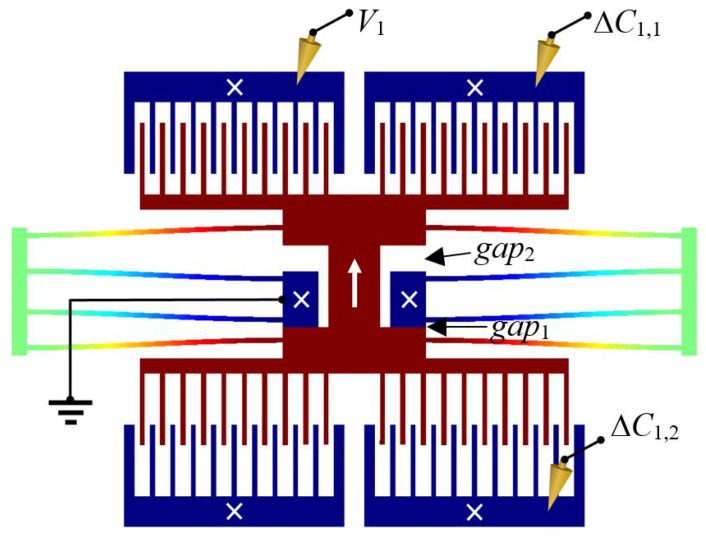
Simulated illustration of an electrically-probed calibration of a MEMS device. One probe applies a voltage *V*_1_ to close one of two asymmetric gaps, while the differential capacitance to traverse *gap*_1_ is measured. Many other aspects of the design, such as the type flexures, are irrelevant. By closing the gaps and resonating the structure, the device’s mass, damping, stiffness, and state can be accurately and precisely measured after packaging and long-term dormancy. This is, it is capable of self-calibration.

*Example*. Let’s apply the above EMM concepts to the type of MEMS shown in Figure 1. The device consists of two pairs of comb drives for actuation and sensing, a pair of asymmetric gaps, and a pair of folded flexure spring supports. Upon fabrication, packaging, and deployment, the device would be subjected to process variations, packaging stress, and environmental changes, yielding the following unknown properties: Young’s modulus, Poisson’s ratio, viscosity, geometric overetch, layer thickness, material density, permittivity, fringing field factor, curvature of radius for the fillets located at all vertices, gaps between comb fingers, etc. As a consequence, the quantities that depend on such properties are unknown as well. This includes mass, damping, stiffness, force, displacement, etc.

The first step is to find an EMM expression $G_{1}^{*}$ that relates an unknown mechanical quantity to electrical quantities. For our testcase, we choose our first seed relation to be overetch. A variety of methods can be used to close gaps, depending on gap size, such as DC voltage, resonance, mechanical force, etc. Overetch can then be used to determine displacement (*G*_2_), which will be used to determine force (*G*_3_), which will be used to determine stiffness (*G*_4_), and so on. As derived in Reference [2], geometric overcut can be determined by measuring the change in capacitances ($\Delta C_{1}$ and $\Delta C_{2}$) required to close the manufactured gaps (*gap*_1_ and *gap*_2_ in Figure 1). That is:

(3) $$gap_{1}=gap_{1,Layout}+\Delta gap=gap_{1,Layout}\left(1-\frac{n+\Delta C_{2}/\Delta C_{1}}{1+\Delta C_{2}/\Delta C_{1}}\right)$$

where Δgap [m] is overcut, i.e., the difference in going from layout gap $gap_{1,Layout}$ to manufactured gap $gap_{1}$; and n≠1 is the ratio between the gaps, i.e., $gap_{2,Layout}=n\;gap_{1,Layout}$. This first measurement of a mechanical quantity is a function of electrical measurements and exactly-known design parameters. It is important to note that the change in capacitance $\Delta C_{1}=\left[C_{1}^{comb}\left(gap_{1}\right)+C_{1}^{P}\right]-\left[C_{1}^{comb}\left(0\right)+C_{1}^{P}\right]$ cancels out the parasitic capacitance $C_{1}^{P}$, which enables this method to be reliable because parasitic capacitance will be different for each comb drive and between each test setup (e.g., dielectric charging, probe contact area, cable orientation, etc.). The well-defined gap-stops enable the method to be repeatable. And utilizing precise electrical measurands enables high accuracy and low uncertainty. For instance, Analog Devices (ADI, Norwood, MA, USA) reports to have sensed an average comb drive displacement of 100 femtometers (a thousandth of the diameter of a hydrogen atom) due to a change in comb drive capacitance of a zeptofarad [23]. Such a sensitivity ratio (comb drive displacement to change in capacitance) agrees with our results below.

The result from Equation (3) can be used to measure the next mechanical quantity, displacement. Since the comb drives will be operating within their linear range, then the ratio of change in capacitance to the distance traversed is the comb drive constant $\Psi_{1}=\Delta C_{1}/gap_{1}$ [F/m]. Therefore, for any intermediate deflection, *x* < *gap*_1_, that produces a change in capacitance ΔC(x), the deflection can be measured as:

(4) $$x=\Delta C\left(x\right)/\Psi_{1}\;[m].$$

The linear operating range also permits us to equate $\partial C_{1}/\partial x=\Delta C_{1}/\Delta x=\Psi_{1}$. Therefore, the drive force, generated by an applied voltage *V*_1_, which includes variable comb finger gaps and fringing fields, is:

(5) $$F_{dr}=\frac{1}{2}\Psi_{1}V_{1}^{2}\;[N].$$

The ratio of the change in force to change in displacement is a measure of stiffness, which includes nonlinearities, defects, fillets, non-rectangular cross sections, course sidewalls, variable widths, variable Young’s modulus, anchor compliance, etc. The stiffness of the system is:

(6) $$K=\frac{1}{2}\Psi_{1}^{2}V_{1}^{2}/\Delta C\left(x\right)\;[N/m].$$

Once stiffness is known, a measure of system mass can be obtained by measuring undamped displacement resonance frequency. However, since a high vacuum is necessary for undamped displacement-resonance frequency, a much more practical measure can be done by measuring the velocity-resonance frequency $\omega \left({\dot{x}}_{\max}\right)=\omega_{0}=\sqrt{K/M}$, which is independent of damping, unlike displacement-resonance. Since mass, stiffness, and velocity-resonance is related by $M=K/\omega_{0}^{2}$, from Equation (6) we have:

(7) $$M=\frac{1}{2\;}\frac{\Psi_{1}^{2}\;V_{1}^{2}}{\Delta C_{1}\;\omega_{0}^{2}}\;[\mathrm{kg}].$$

Damping *D* is related to mass by $D=2M\gamma^{2}$, where the exponential decay rate is $\gamma =\sqrt{\left(\omega_{0}^{2}-\omega_{r}^{2}\right)/2}$ and $\omega_{r}$ is the damped displacement-resonance frequency. We therefore have:

(8) $$D=\sqrt{\frac{\omega_{0}^{2}-\omega_{r}^{2}}{2}}\frac{\Psi_{1}^{2}\;V_{1}^{2}}{\omega_{0}^{2}\;\Delta C_{1}}\;[N\cdot s/m].$$

At this point in our sequence of quantity extractions, we now have quantities that define the microsystem’s equation of motion along the *x*-direction:

(9) $$M\ddot{x}+D\dot{x}+Kx=F_{dr}+\sum F_{external}$$

Continuing on, the sequence of quantity extractions leads to relations for displacement amplitude, quality factor, Young’s modulus, material density, viscosity, layer thickness, etc. For example, amplitude and qualify factor are:

(10) $$x_{\max}=\frac{F_{\max}}{D\sqrt{\left(\omega_{0}^{2}-\omega_{r}^{2}\right)/2}}\;[m]$$

and:

(11) $$Q=\omega_{d}^{}\frac{M}{D}=\frac{1}{2}\sqrt{\frac{\omega_{0}^{2}+\omega_{r}^{2}}{\omega_{0}^{2}-\omega_{r}^{2}}}$$

where $\omega_{d}^{}=\sqrt{\left(\omega_{0}^{2}+\omega_{r}^{2}\right)/2}$ is the exponentially decaying oscillation frequency without drive excitation ($F_{dr}=0$).

So instead of being limited to frequency response, this metrological method enables accurate measurements of mass, damping, stiffness, force, and state of the system. In particular, this facilitates accurate sensing of force and displacement due to external disturbances, which can be quite useful since nearly all physical quantities can be traced to relations that depend on force and displacement.

As Equation (2) describes, measurements of uncertainties for each of the extracted quantities of (3) to (11) can be determined by a truncated multivariate Taylor expansion, where each electrical measurand is expressed in terms of its nominal and uncertain quantities; i.e., $e_{measured}\to e\pm \delta e$. For instance, for capacitance and voltage, we have:

(12) $$\begin{array}{ll} \Delta C_{measured} & =\left(C_{final}\pm \delta C\right)-\left(C_{initial}\pm \delta C\right)\\ & =\left(C_{final}-C_{initial}\right)\pm \sqrt{\sum_{i=1}^{2}{\left(\delta C_{i}\right)}^{2}}\\ & =\Delta C\pm \sqrt{2}\;\left|\delta C\right|\;[F] \end{array}$$

and similarly:

(13) $$V_{measured}=V\pm \sqrt{2}\;\left|\delta V\right|\;[V],$$

where it is assumed that δC and δV represent the totality of all noise, which can be conservatively determined by the decimal place of the most significant uncertain digit on, say, the capacitance or voltage meter’s readout display. For example, the uncertainty expressions for displacement (4) and force (4) are:

(14) $$\delta x=\left\{\sqrt{2}\;gap_{1,Layout}\frac{\left(n-1\right)\left(2\Delta C\left(x\right)-\Delta C_{1}-\Delta C_{2}\right)}{{\left(\Delta C_{1}+\Delta C_{2}\right)}^{2}}\right\}\delta C$$

(15) $$\delta F=\left\{\frac{\sqrt{2}\;V}{gap_{1,Layout}\left(n-1\right)}\right\}\delta C+\left\{\frac{\sqrt{2}\;\left(\Delta C_{1}+\Delta C_{2}\right)}{gap_{1,Layout}\left(1-n\right)}\right\}\delta V$$

where the curly-bracketed expressions are the sensitivities. For the device in Figure 1, the sensitivity of δx is on the order of $O\left(10^{9}\right)$ meters/Farad for the device in Figure 1, so an uncertainty for δx that would be on the order of an angstrom requires that $\delta C=O\left(10^{-10}\right)$ [F].

Off-the-shelf capacitance meters can have precisions of $\delta C=O\left(10^{-12}\right)$F, or a pF [24,25,26]. The most precise capacitance meters to date have $\delta C=O\left(10^{-21}\right)$F, or zF [27,28]. Similarly, the sensitivities of δF are $O\left(10^{6}\right)$ and $O\left(10^{-9}\right)$ for the first and second terms in Equation (15). The order of precision for voltage source/meters typically range from millivolts to nanovolts. Such a range of precisions for capacitance and voltage are expected to result in a range of force uncertainties from micronewtons to femtonewtons. However, there are also other ways to improve precision. In Reference [21] it is shown that the order of EMM uncertainties also depends on design parameters, which affect the order of sensitivities for δx and δF. That is, there will be a decrease in sensitivity if there is a decrease in comb finger gap *g_f_*, or if there is an increase in the number of comb fingers *N_f_*, gap ratio *n*, layer thickness *h*, or gap-stop size *gap*_1_. So by choice of device design parameters and meter precisions, a desired precision magnitude may be achieved. Tangible examples of force magnitudes are provided in Table 1.

## 3. Performance Control

The previous section described how system mass, damping, stiffness, force, and state can be accurately and precisely measured using electro micro metrology (EMM), this section describes how the effective mass, damping, and stiffness of a MEMS device can be changed, such that the microsystem can modify its performance on demand (POD). EMM can be used to recalibrate to the modified effective mass, damping, and stiffness of a PODMEMS device.

**Figure 2:**
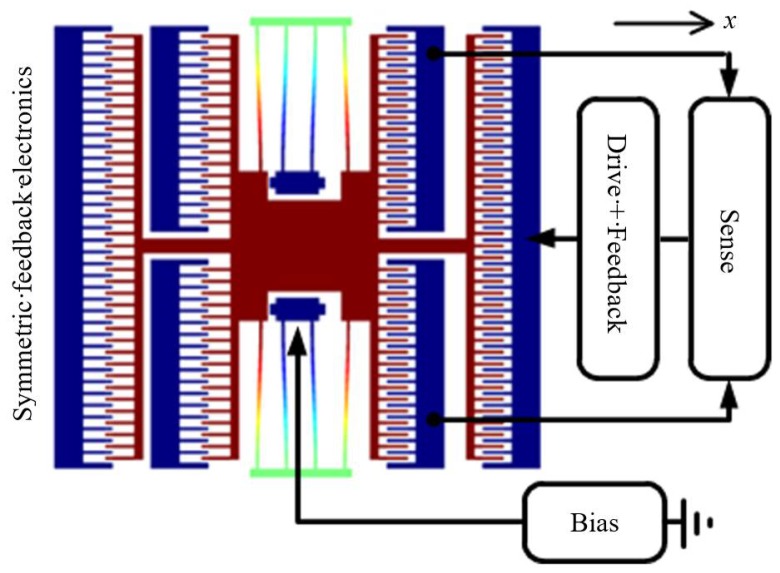
MEMS + performance controller [29]. Symmetric feedback components on the right and left (not shown) sides of the structure operate 180° out of phase for continuous feedback response throughout each cycle. Feedback forces are applied to left and right combs according to position, velocity, and acceleration.

With control over effective mass, damping, and stiffness, PODMEMS devices are expected to be able to compensate for performance variations due to processing, packaging, temperature, noise, damping, or extend dynamic range, or be used to accommodate multiple application modes. Such control is achieved by continuously monitoring the state of the proof mass, and feeding back forces $F_{fb}\left(x^{\tau },{\dot{x}}^{\tau },{\ddot{x}}^{\tau }\right)$ onto the proof mass that are proportional to displacement, velocity, and or acceleration. A system-level view of the feedback for a PODMEMS device is shown in Figure 2. With the addition of feedback forces $F_{fb}$, the equation of motion (9) becomes:

(16) $$\begin{array}{ll} M\ddot{x}+D\dot{x}+Kx & =F_{dr}-\left[F_{fb}\left(x^{\tau },{\dot{x}}^{\tau },{\ddot{x}}^{\tau }\right)\right]\\ & =F_{dr}-\left[F_{K}+F_{D}+F_{M}\right]\\ & =F_{dr}-\left[K_{e}x^{\tau }+D_{e}{\dot{x}}^{\tau }+M_{e}{\ddot{x}}^{\tau }\right] \end{array}$$

where $F_{fb}=F_{K}+F_{D}+F_{M}$; the state-proportional terms are $F_{K}=K_{e}x^{\tau }$, $F_{D}=D_{e}{\dot{x}}^{\tau }$, and $F_{M}=M_{e}{\ddot{x}}^{\tau }$; and the sensed displacement $x^{\tau }=x\left(t-\tau \right)$, velocity ${\dot{x}}^{\tau }=\dot{x}\left(t-\tau \right)$, and acceleration ${\ddot{x}}^{\tau }=\ddot{x}\left(t-\tau \right)$ of the proof mass are subject to feedback delay τ due to the RC time constant of the analog feedback circuit. The quantities *K_e_*, *D_e_*, and *M_e_* are the electrically-generated proportionality constants that can increase or decrease the effective mass, damping, or stiffness system. That is, Equation (16) may be rewritten as:

(17) $$\begin{array}{ll} F_{dr} & =\left(M\ddot{x}+M_{e}{\ddot{x}}^{\tau }\right)+\left(D\dot{x}+D_{e}{\dot{x}}^{\tau }\right)+\left(Kx+K_{e}x^{\tau }\right)\\ & =M_{eff}\ddot{x}+D_{eff}\dot{x}+K_{eff}x \end{array}$$

where the effective mass, damping, and stiffness may be expressed as:

(18) $$\begin{array}{c} M_{eff}\approx M+M_{e}-D_{e}\tau \\ D_{eff}\approx D+D_{e}-K_{e}\tau +M_{e}\omega^{2}\tau ,\\ K_{eff}\approx K+K_{e}, \end{array}$$

assuming low latency. For large latencies, exact expressions for $M_{eff}$, $D_{eff}$, and $K_{eff}$ are derived in [29]. As seen in Equation (18), there is a small amount of crosstalk within the effective mass and damping, i.e., $D_{e}\tau$, $K_{e}\tau$, $M_{e}\omega^{2}\tau$, where the amount of crosstalk is reduced by feedback delay.

**Figure 3:**
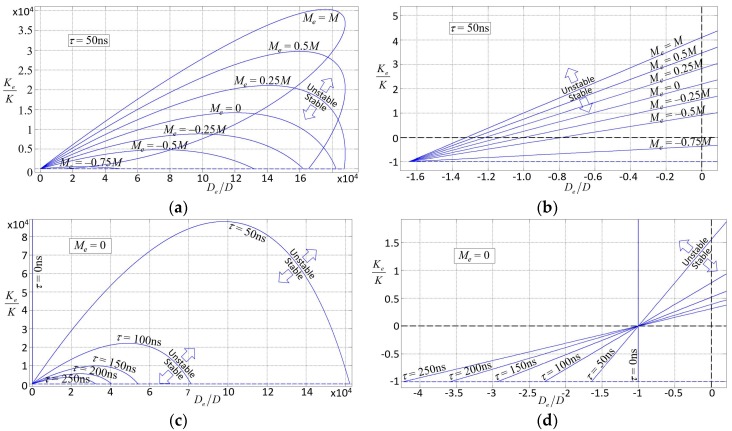
Domains of stability for PODMEMS [29]. The horizontal-axis is the ratio of electrical to mechanical damping. The vertical-axis is the ratio of electrical to mechanical stiffness. The dome areas between the curve and horizontal line *K_e_/K* = −1 are domains of stability. Within a dome, stability decreases the closer the state is to its boundary. Stability is zero on the boundary. And outside a dome, instability increases the farther the state is from its stability boundary. The plot in (**a**) shows stability domains for different values of *M_e_*. (**b**) is a magnified view about point (*D_e_/D*, *K_e_/K*) = (0, 0); (**c**) shows stability domains for different values of *τ*; and (**d**) shows a magnified view near (0,0). Note, further magnification of (**d**) shows that all curves do not intersect at a single point near (−1, −2.9 × 10^−3^).

The dynamical range of PODMEMS is much greater than what can be achieved by MEMS that do not exploit feedback. Due to feedback latency, instability is also possible. The dynamic range and stability of a PODMEMS device can be visually determined by Figure 3a–d as follows (exact expressions for stability are derived in Reference [29]). The vertical and horizontal axes in Figure 3 are *K_e_*/*K* and *D_e_*/*D*. The family of curves (or lobes) indicate the bounds of instability, where PODMEMS is stable within the lobe, which is bounded between the curve and dashed line at *K_e_*/*K* = −1. PODMEMS is unstable outside a given lobe. The family of lobes are parameterized by *M_e_* in Figure 3a,b, and parameterized by delay *τ* in Figure 3c,d. Interesting aspects of Figure 3 are that: (1) most MEMS, which are passive, are constrained to operate at the origin, at (*K_e_*/*K*, *D_e_*/*D*, *M_e_*/*M*) = (0, 0, 0), while PODMEMS may operate throughout the entire 3D performance control space of (*K_e_*/*K*, *D_e_*/*D*, *M_e_*/*M*); (2) as shown in Figure 3d, there are regions where the overall damping can be negative while stability is maintained; (3) the electrical mass, damping, and stiffness are allowed to be several orders greater in magnitude than their purely mechanical counterparts; and (4) due to the small length scale and time scale of MEMS, the domain of stability for any PODMEMS is very sensitive to feedback latency.

With a system described by Equation (17), the characteristic effective exponential decay rate $\gamma_{eff}$, quality factor $Q_{eff}$, amplitude at displacement resonance $x_{\max}$, and velocity resonance $\omega_{0}$ are:

(19) $$\gamma_{eff}=\frac{1}{2}D_{eff}/M_{eff}$$

(20) $$Q_{eff}=\frac{\omega_{d,eff}}{2\gamma_{eff}}$$

(21) $$x_{max}=\frac{F_{dr}}{D_{eff}\omega_{d,eff}}$$

(22) $$\omega_{0,eff}=\sqrt{\frac{K_{eff}}{M_{eff}}}$$

(23) $$D_{eff}\;\{\;\begin{array}{l} <\sqrt{4M_{eff}K_{eff}},\;\text{under-damped}\\ =\sqrt{4M_{eff}K_{eff}},\;\text{critically-damped}\\ >\sqrt{4M_{eff}K_{eff}},\;\text{over-damped} \end{array}$$

where, instead of such characteristic behaviors being constant as they are for passive MEMS, such characteristics for PODMEMS are greatly modifiable, controlled by electrical feedback forces that depend on real-time state monitoring.

Let’s apply the above PODMEMS concepts to the MEMS device shown in Figure 2. The design parameters for the device are: pairs of folded flexures having width *w* = 2 μm, thickness *h* = 20 μm, and length *L* = 294.7 μm; pairs of 100-finger comb drive arrays with finger length *L_f_* = 20 μm, finger width *w_f_* = 2 µm, and gap *g_f_* = 2 μm; Young’s modulus *E* = 160 GPa, density *ρ* = 2300 kg/m^3^, structure-to-substrate gap *g_gnd_* = 2 μm, viscosity *μ* = 1.75 × 10^−5^ sPa, and proof mass area *a_m_* = 17,424 μm^2^ (including flexures and combs). This yields a lumped mass, damping, stiffness, and nonlinear stiffness of *M* = *ρ* × volume = 8 × 10^−10^ kg, *D* = $\mu a_{m}/g_{gnd}$ = 1.55 × 10^−7^ Ns/m, *K* = 2*Ehw*^3^/*L*^3^ = 2 N/m, and *K_NL_* = *K* + *κx*^2^, where *κ* = π^2^*Ewh*/(64*L*^3^) = 4.10 × 10^10^ N/m^3^. The purely mechanical period of the structure is 12.57 μs. Specifications of off-the-shelf electronics for simple feedback yields a delay time of *τ* ≈ 50 ns.

When designing feedback control systems, it can be useful to express maximal mechanical forces in terms of equivalently-applied voltages; i.e., the voltage necessary to generate the equivalent feedback force. For simple harmonic motion, we have:

(24) $$V_{K}^{}=\sqrt{Kx_{\max}/\frac{1}{2}\Psi }$$

(25) $$V_{D}^{}=\sqrt{D\omega_{r}x_{\max}/\frac{1}{2}\Psi }$$

(26) $$V_{M}^{}=\sqrt{M\omega_{r}^{2}x_{\max}/\frac{1}{2}\Psi }$$

where the applied control voltages $V_{K}$, $V_{D}$, and $V_{M}$, correspond to feedback forces $F_{K}$, $F_{D}$, and $F_{M}$. For example, using the PODMEMS device from Figure 2 where $\omega_{r}=50\;\mathrm{krad}/\sec$ and $\Psi \approx 2N_{f}\varepsilon h/g_{f}=3.5\times 10^{-14}\;F/m$, for $x_{\max}=0.1\;\mu m$, we have $V_{D}=0.66\;V$ and $V_{K}=V_{M}=10.6\;V$; or for $x_{\max}=1\;\mu m$, we have $V_{D}=2.1\;V$ and $V_{K}=V_{M}=33.6\;V$. That is, control voltages can be reduced by decreasing $x_{\max}$, *K*, or *D*, or by increasing Ψ or *M*. Ψ can be increased by decreasing finger gaps size *g_f_*, or increasing layer thickness *h*, or number of fingers *N_f_*.

For example, let’s apply the above analysis to halving or doubling the resonance frequency of a MEMS device. This can be done by decreasing $K_{eff}$ or $M_{eff}$ such that $\omega_{0,eff}=\sqrt{K_{eff}/M_{eff}}=\frac{1}{2}\sqrt{K/M}$ or $2\sqrt{K/M}$. This involves setting $K_{eff}=K+\left[K_{e}^{}\right]=K+\left[-3K/4\right]=K/4$ to halve the resonance or $M_{eff}=M+\left[M_{e}^{}\right]=M+\left[-3M/4\right]=M/4$ to double the resonance of its purely mechanical counterpart.

## 4. Possible IoT Applications

This section envisions a few possible applications of self-calibration and performance control that may be useful for IoT and other application areas.

### 4.1. Metrology

The benefits of self-calibration are expected to: (1) reduce the cost of MEMS devices since the costly expense of in-factory calibration can be reduced or eliminated, which should also increase manufacturing throughput; (2) greatly extend the usefulness of sensors since devices will be able to re-calibrate after long-term dormancy or after harsh environmental change; (3) improve the quality of data being analyzed in terms of increased accuracy and reduced uncertainty; and (4) close the large gap in going from experiment back to simulation, to build experimentally-accurate predictive computer models of IoT sensors and of their environments. Examples follow:

*Temperature sensors*. Accurate measurements of temperature can be useful for improved: weather predictions, ecological health monitoring, infrared sensing, sensing of factory equipment or chemical processes, implantable health monitoring, continuous measurement of engine efficiency, heat energy, pressure, volume, thermal efficiency, coefficient of performance, entropy production, etc. Due to the length scale, most microdevices are subject to measurable thermally-induced vibrations. From the equipartition theorem, mechanical potential energy is related to thermal energy by $\frac{1}{2}K\langle x^{2}\rangle =\frac{1}{2}k_{B}T$ [30] where $k_{B}$ is the Boltzmann constant, T is absolute temperature, and $\langle x^{2}\rangle$ is the mean-square of displacement due to thermally-induced vibrations, and other sources. Since EMM enables accurate measurements of flexure stiffness *K* and displacement *x*, then by the equipartition theorem, the absolute temperature T of the MEMS device can be accurately measured.

**Figure 4:**
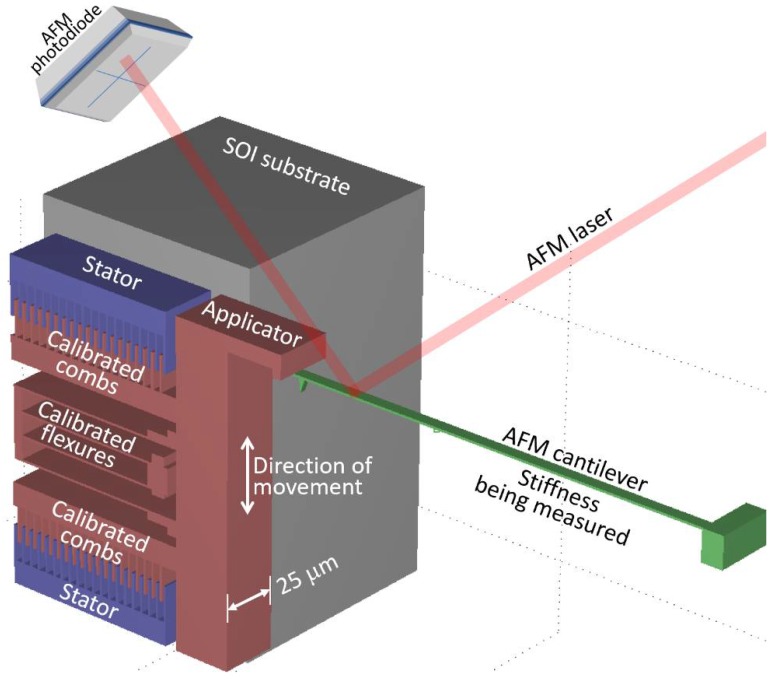
Illustration of AFM calibration. A calibrated MEMS is used to measure the stiffness of an AFM cantilever. The photodiode may be simultaneously calibrated to the cantilever’s deflection.

*Atomic force microscopy and biosensors*. Once a MEMS device is accurately calibrated, it becomes an accurate sensor for calibrating other devices, such as cantilevers for atomic force microscope (AFM), cantilevers for biosensors, the self-calibration of AFM-on-chips, mass sensors, etc. See Table 1. Due to the inaccuracies and large uncertainties (>10%) of conventional cantilever stiffness measurement methods [30,31,32,33,34], it is difficult for biotechnologists to discern targeted from nonspecific bindings, and most AFM users do not use the AFM to measure force [35,36,37]. However, by pressing the tip of a cantilever against the sidewall of a calibrated MEMS device (Figure 4), the MEMS device can apply a known force (5) to deflect the cantilever by measured amount (4). The force balance relation would be $F=\left(K+K_{AFM}\right)x$, where $K_{AFM}$ is the unknown AFM cantilever stiffness to be determined. Since $x_{AFM}=x$, then the photodiode can be simultaneously calibrated.

*Mass sensors*. Depending on application, a mass m can be adsorbed onto a calibrated vibrating diaphragm, or m can be measured within a microfluidic channel embedded within flexures and proof mass. Although nano- to micro-mechanical resonant mass sensors have been able to detect a change in frequency due to additional mass *m*, the accurate measurement of that added mass has been difficult due to the inability to accurately measure the system stiffness *K* and mass *M* [38,39,40]. The ability of EMM to accurately measure MEMS stiffness *K* and mass *M* can be used to measure the added mass m of a resonating device; i.e., $\omega_{0}^{}=\sqrt{K/\left(M+m\right)}$, where m is the unknown to be determined.

*Gravimetry*. Gravimeters are often used to measure changes in the earth’s gravitational field due to large underground deposits of resources, human activities, or seismology. The accuracy of relative or absolute MEMS-based gravimeters [41,42] depend on accurate calibration of stiffness and mass. By tilting an EMM-calibrated MEMS device from being perpendicular to parallel to the direction of gravitational acceleration, then absolute gravity can be measured as $K\;x=M\;{\ddot{x}}_{g}$, where ${\ddot{x}}_{g}$ is the unknown gravitational acceleration being measured.

*Altimetry*. Conventional MEMS altimeters work by measuring atmospheric pressure [43,44]. The measurement of height can be an additional dimension added to GPS. As a MEMS device changes in height *h_g_*, there is a small change in gravity. The change in gravitational force on a MEMS proof mass of, say, M= 10 × 10^−9^ kg, by the height of a person, the Empire State Building, or the cruising altitude of a passenger jet is 4 fN, 1 pN, or 26 pN, as determined by Newton’s law of gravitation: $\Delta F=G\;M_{Earth}\;M\left[R_{Earth}^{-2}-{\left(R_{Earth}+h_{g}\right)}^{-2}\right]$, where *G* = 6.67 × 10^−11^ $m^{3}\cdot {\mathrm{kg}}^{-1}\cdot s^{-2}$, $M_{Earth}$ = 5.9 × 10^24^ kg, $R_{Earth}$ = 6.37 × 10^6^ m. Or, if gravity is assumed to be constant, the change in height $\Delta h_{g}$ can be determined by equating gravitational potential energy to the mechanical potential energy stored in the flexure: $M\;{\ddot{x}}_{g}\;\Delta h_{g}=\frac{1}{2}K_{}\left(x_{}^{2}-x_{reference}^{2}\right)$.

*Inertial measurement units*. More accurate translational and or rotational reckoning may be required by mobile IoT devices such as drones, self-driving vehicles, robots, precision handheld tools, automated surgical tools, manufacturing machines, refined GPS (<<1 m), air/marine/space craft, etc. Present IMUs have difficulty with drift due to thermal expansion from changes in temperature [45]. EMM can be used to recalibrate IMUs as temperatures change, and because the equation of motion is known, the forces due to movement within a non-inertial reference frame can be accurately measured. These forces include: (1) the Coriolis force due to the proof mass M moving with velocity $\dot{r}=\dot{x}$ in a frame that is rotating with frequency vector ω: $F_{Coriolis}=-2M\;\omega \times \dot{r}$; (2) the Euler force due to M located at a displacement vector r from the point of rotation of a nonconstant frequency vector $\dot{\omega }$ that is changing in magnitude and or direction: $F_{Euler}=-M\;\dot{\omega }\times r\;;$ (3) the centrifugal force on $M_{EMM}$ along the direction of vector r from the point of rotation vector ω to M: $F_{centrifugal}=-M\;\omega \times \left(\omega \times r\right)$; and (4) the translational force due to the acceleration $\ddot{R}$ of the sensor’s chip (or frame of reference): $\;F_{translational}=-M\;\ddot{R}$. Since a self-calibrated IMU would be able to directly measure these non-inertial forces, then the respective unknowns (ω, $\dot{\omega }$, r, $\ddot{R}$) become measurable.

*Pressure sensors/Sound sensors*. Conventional MEMS pressure sensors or microphones are usually based on piezo or capacitive transduction [46,47,48]. Pressure sensors typically consist of a diaphragm that is exposed to external pressures and substances. The diaphragm of implantable pressure sensors for blood pressure monitoring can become coated with biomatter, which changes the diaphragm’s effective stiffness. Using a vertical comb drive to sense diaphragm deflection, the diagram may be re-calibrated to its new stiffness prior to measurement. Additionally, if an implantable sensor consisted of a pair of calibrated pressure sensors at a known distance apart, then in addition to absolute pressure, the viscosity and velocity of blood flow may also be measured.

*Diagnostic structure/Quality control*. Examples of prior efforts in MEMS diagnostics include using large arrays of test structures [49] and coupled measurement plus computer modeling [50]. Once a device’s mass, damping, and stiffness are measured, continuing on with the sequence of property extractions, geometric and material properties can be determined as well. In this way, an EMM diagnostic chip can accompany each process run for quality control purposes. Since EMM can be electrically probed, the testing and corresponding modification of the fabrication recipe can be automated. The diagnostic chip may also be placed at different locations about the wafer to examine how, say, Young’s modulus, varies across the wafer, from wafer to wafer, from run to run. For MEMS devices that are not amenable to direct EMM calibration, an EMM test structure device can be manufactured alongside the main device, within close-proximity, such that the main IoT sensor and the EMM test structure would be subjected to closely-matched geometric and material property variations.

*Experimentally-accurate modeling*. The performance of a fabricated MEMS device diverges significantly from its originating CAD model due process variations and other nonidealities. By substituting a device’s measured parameters into the CAD model, higher-order effects can be studied for a better understanding of the device behavior. Improved understandings can enable the causes of subtle disturbances to be more easily identified or can pinpoint areas of improvements for the next design generation to shorten design cycles. This can ultimately extend to the refined modeling of distributed IoT phenomena such as environmental models.

### 4.2. Performance Control

The benefits of performance control are expected to: (1) close the large gap in going from simulation to experiment, whereby the device corrects for variations in processing, packaging, and environment; (2) optimize sensing behavior to environmental conditions; (3) extend utility and dynamic range beyond the limits of structural micromachining constraints; and (4) maintain or improve manufacturing yield while improving performance.

**Figure 5:**
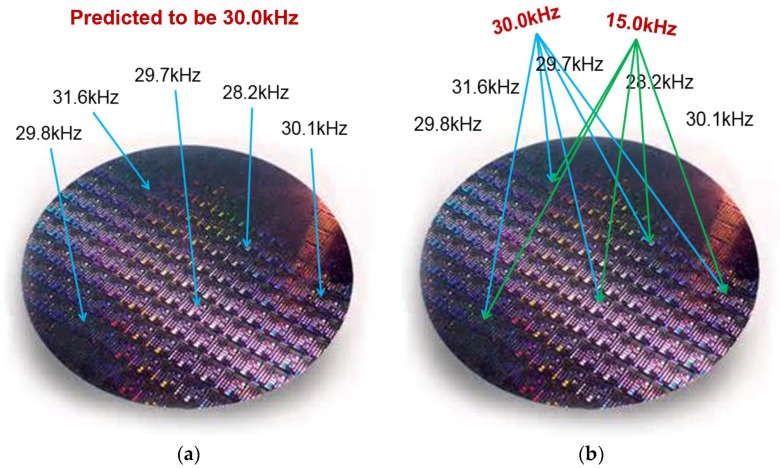
An illustration of PODMEMS resonance control for a multitude of resonators that were initially designed to resonate at 30.0 kHz. (**a**) Due to process variations, none of the MEMS devices will naturally resonate at 30.0 kHz. (**b**) By activating PODMEMS performance control, all devices will be able to identically resonate at 30.0 kHz, or change resonance on demand to a different frequency, such as 15.0 kHz.

*Resonance control*. MEMS sensors often utilize resonance for stronger signal to noise ratios, filtering, signal processing, timing, inertial navigation, communications, etc. As previously mentioned, prescribing a particular resonance is problematic due to process variations and requires tuning [51]. As illustrated in Figure 5a, a wafer of identically laid out resonators that were predicted to have a particular resonance frequency within CAD will differ in resonance upon fabrication. However, by adjusting the effective mass or stiffness by electrical force feedback, the desired resonance can be obtained. Moreover, the resonances of all devices can change to a different value on demand (see Figure 5b). For example, the resonance can be reduced by a factor of two by reducing the effective stiffness by a factor of four. That is, by setting the feedback electrical stiffness to be $K_{e}=-\frac{3}{4}K$, then the new resonance becomes $\omega_{0,new}=\sqrt{K_{eff}/M}=\sqrt{\left(K+K_{e}\right)/M}=\sqrt{\frac{1}{4}K/M}=\frac{1}{2}\omega_{0,old}$.

*Frequency locking*. Drift is a significant challenge for devices that rely on a particular resonance frequency, such as IMUs, clocks, and notch filters. Small shifts in resonance frequency from drift can result in significant amplitude attenuation due to extremely narrow bandwidths. Besides tracking frequency [52,53], it may be possible to counter drift by correcting changes in the effective stiffness in real time to maintain a constant resonant frequency. For instance, since MEMS resonance (i.e., stiffness) drifts with temperature, K(T), the MEMS device can be regarded as the slave-oscillator of a phase locked loop, where the constant driving frequency is the master-oscillator. A phase detector measures the phase between the drifting MEMS frequency and the constant driving frequency. The phase signal is then amplified, filtered, and can be used to increase or decrease the electrical feedback stiffness $K_{e}^{PLL}$, which counters the drifted frequency of the MEMS device, maintaining a desired frequency $\omega_{0}=\sqrt{\left[K\left(T\right)\pm K_{e}^{PLL}\right]/M}=const.$, which effectively locks the device to a constant frequency that is independent of temperature.

*Mode matching*. The ability to match or follow frequencies can be beneficial. For instance, the small Coriolis force that is proportional to the velocity of the primary mode in vibratory gyros is what drives the secondary mode to amplitudes that are often on the order of nanometers. The size of the amplitude depends on how well the primary and secondary resonance modes are matched. Matching can also eliminate *blind spots*. Blinds spots in vibratory gyros happen when the velocity in the primary mode nears the turning points of oscillation, when velocity becomes too small to produce a significant Coriolis force for the secondary mode, $F_{Coriolis}=-2M\;\omega \times {\dot{r}}_{1}$. That is, $F_{Coriolis}\to 0$ as ${\dot{r}}_{1}\to 0$ at the turning points of oscillation, regardless of the size of the input disturbance ω during that moment. Vibratory gyros experience such blindness twice per period, which contributes to inaccurate results. However, a pair of matched gyros that maintain a 90° phase between them should able to eliminate blind spots, whereby one gyro will have a peak velocity whenever the other has a zero velocity, such that either velocity is significant at all times.

**Figure 6:**
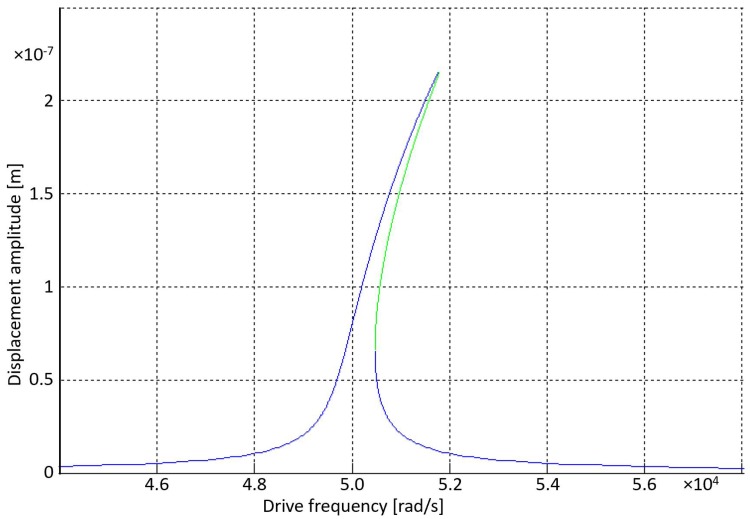
Nonlinear frequency response of $M\ddot{x}+D\dot{x}+Kx+\left(\kappa +\kappa_{e}\right)x^{3}=F_{0}\cos\left(\omega t\right)$, where $M=8\times 10^{-10}\;\mathrm{kg}$, $D=1.55\times 10^{-7}\;\mathrm{Ns}/m$, K=2 N/m, $\kappa =4\times 10^{10}\;N/m^{3}$, $\kappa_{e}=4\times 10^{12}\;N/m^{3}$, and $F_{0}=1.7\times 10^{-9}\;N$ due to a driving voltage of $V_{0}=0.9\;V$. Note that $\kappa_{e}$ is two orders larger than the purely mechanical κ, which enables the device to experience high nonlinearity at a much smaller amplitude, which would otherwise result in a linear response. The benefit of an early onset of nonlinearity enables large amplitudes to be maintained during resonance drift; i.e., left (or right) translation of the response curve due to an increase (or decrease) in temperature.

*Nonlinear dynamics*. Geometric nonlinearity in stiffness affects the linearity of sensors. Such nonlinearity can be reduced or increased by a feedback force of the Duffing-type $F_{\kappa }=\pm \kappa_{e}{\left(x^{\tau }\right)}^{3}.$ Such control can be used to increase the linearity of sensors or to introduce nonlinearity to linear (small-deflection) devices [20]. The early onset of nonlinearity can be used to maintain a large resonance amplitude during frequency shifts by spring hardening or softening. For example, a feedback voltage of V=4.5 V at an amplitude of $x=2.2\times 10^{-7}\;m$ is equivalent to $\kappa_{e}=4\times 10^{12}\;N/m^{3}$, which is two orders greater than the purely mechanical geometric stiffness (see Figure 6). If the driving frequency is detuned to ω=51.5 krad/s, instead of driving at $\omega =\omega_{0}=50.0$ krad/s, then as the response curve translates to the left due to an increase in temperature of, say, $100{\;}^{o}C$, then the displacement amplitude would only change by about 0.02%. However, if the response curve was that of a linear high-*Q* device (vertical pole with an extremely narrow bandwidth), then a slight shift in resonance would experience significant attenuation in displacement amplitude.

*Displacement noise reduction*. An increase in effective damping can be useful for static measurements, reduction of transients, or nanoscale manipulation or positioning beyond the thermal noise limit [17,18]. For static measurements, systems that are effectively critically-damped offer the fastest route to static equilibrium by setting $D_{eff}$ to be $\sqrt{4M_{eff}K_{eff}}$. This is done by feeding back a force that is proportional and opposite to sensed velocity. A reduction in displacement noise by damping could also benefit applications that would otherwise require a significant amount of averaging. 

*High Q*. Quality factors *Q* are critically important for gyro sensitivity (bias °/h). Applications for gyro sensitivity range from low-end tactical (1°/h < bias < 15°/h, 1000 < *Q* < 10,000), tactical (0.1°/h < bias < 1°/h, 10,000 < *Q* < 40,000), to navigational (0.001°/h < bias < 0.1°/h, 40,000 < *Q* < 10M). As *Q* increases from 1000 to 10 M, cost increases from $10 to $100k. A decrease in effective damping *D_eff_* should be useful for achieving a higher effective quality factor *Q_eff_*, whereby the energy lost per cycle is fed back into the system. Achieving high-*Q_eff_* through feedback should be less costly. Usually, the higher the *Q*, the more difficult it is to match driving frequency with device resonance frequency due to drifting temperatures due to a narrowing vertical bandwidth. However, as modeled in Figure 6, nonlinear feedback may be viewed as effectively increasing the bandwidth, not by increasing the width of the curve, but by controllably bending the curve to the right by spring hardening or bending the curve to the left by spring softening. Such nonlinearity effectively increases the frequency range at which the driving frequency results in a large displacement amplitude. This enables high-*Q* resonators to more easily maintain a large displacement amplitude while the resonance drifts by using a detuned driving frequency. Previously, spring softening has been achieved by using small gap-closing electrodes with limited displacement amplitude, and spring hardening has been achieved by exploiting mechanical geometric stiffness due to large displacement amplitudes [54]. 

*Modularity*. Devices that can modify their effective mass, damping, and or stiffness should be amenable to achieving modularity, where a new device should be able to mimic the performance of the old device that it is replacing. Or conversely, a distribution of old sensors may be required to update their performances to that of new sensing specifications.

*Beyond micromachining limits*. It should be possible to significantly reduce the effective mass or stiffness along a particular degree of freedom to that of, say, a nanoscale device. That is, while there are micromachining limits to the flexures and proof masses for MEMS, the effective stiffness or mass can be reduced in the *x*-direction to that of a much smaller device. Since this method of performance enhancement does not require approaching the fragile limits of micromachining, more robust devices can be designed that maintain high manufacturing yields.

## 5. Conclusions

The small size and low power requirements of MEMS sensors are favorable attributes for IoT. IoT applications that require accurate sensing analysis with low uncertainty require the sensed input data to be also be accurate with low uncertainty. Presently, most MEMS devices are inaccurate with large uncertainties. However, we have shown that is it possible to develop MEMS devices that can autonomously calibrate themselves to achieve much higher accuracy and much lower uncertainty. And since uncomprehensive in-factory calibration is time-consuming and bottlenecks throughput, it can increase the cost MEMS devices by as much as 300%. However, self-calibration may be able to reduce this cost by reducing or eliminating the need for in-factory calibration; and since self-calibration is repeatable and reliable, the method may lead to international standardization. Another beneficial attribute of MEMS devices for IoT is their exceptional performance, where they can, for instance, achieve much higher quality factors than purely electrical systems. But performance depends on structural design, which has been constrained by manufacturing limits. However, our results suggest that such constraints may be bypassed by feedback performance control, where performances that were previously intractable could be easily achieved by controlling the effective mass, damping, and stiffness of the system. While these initial studies of self-calibration and performance control utilize the comb drive sensor/actuator, further investigations are needed to extend these methods to the other types of microtransducers. The ability of future microsensors to change their performance behaviors on demand, or to accurately self-characterize themselves, are expected to greatly extend the applications and utility of MEMS for IoT, as well as other areas.

## Figures and Tables

**Table 1:** Relative magnitudes of tangible forces.

Force	Tangible Phenomena	Conventional Tools		
1 N	Weight of Newton’s apple	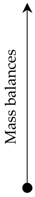		
10^−1^ N	Translational force on a pitcher’s curveball			
10^−2^ N	Width of 1 cm^3^ of water			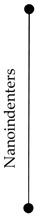
10^−3^ N	Particulates in 1 m^3^ of urban air			
10^−4^ N	Indentations		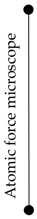	
10^−5^ N	Surface tension per centimeter of water			
10^−6^ N	Solar radiation/m^2^ near earth			
10^−7^ N	Exact force/length of a pair of 1 ampere wires 2 m apart			
10^−8^ N	Weight of a dust mite, or hydrogen/liter of water pH7			
10^−9^ N	Covalent bond			
10^−10^ N	Noncovalent bond, or DNA rupture			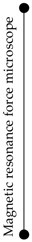
10^−11^ N	Gravitational force between two 1 kg masses 1 m apart	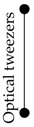		
10^−12^ N	Light pressure of a 1 mW laser pointer, or protein folding			
10^−13^ N	Casimir force/μm^2^ on parallel plates 1 μm apart			
10^−14^ N	Weight of bacterium, or force of its Brownian motion			
10^−15^ N	Resolution of optical tweezers			
10^−16^ N	Force between a pair of electrons 15 μm apart			
10^−17^ N	Resolution of magnetic force resonance force microscopy			
10^−18^ N	Unpaired electron spins			
